# Leprosy

**Published:** 1856-03

**Authors:** 


*Leprosy.*—Dr. R. Stewart, of the Calcutta and Leper Asylum, reports his great success in treating the worst forms of leprosy with the seed of the *chaul moogra.* In a late number of this journal (*ante, p.* 231) we noticed the favorable reports of the American Missionary, at Canton, Dr. Hobson, on the use of this nut in the treatment of a hitherto incurable disease. The Chinese call it *tae fung tsze*, and we may hope to find it valuable in the cure of obstinate skin affections of all varieties.

